# Neurologic Manifestations as Initial Clinical Presentation of Familial Hemophagocytic Lymphohistiocytosis Type2 Due to *PRF1* Mutation in Chinese Pediatric Patients

**DOI:** 10.3389/fgene.2020.00126

**Published:** 2020-03-04

**Authors:** Wei-xing Feng, Xin-ying Yang, Jiu-wei Li, Shuai Gong, Yun Wu, Wei-hua Zhang, Tong-li Han, Xiu-wei Zhuo, Chang-hong Ding, Fang Fang

**Affiliations:** Neurology Department, National Center for Children’s Health China, Beijing Children Hospital affiliated to Capital Medical University, Beijing, China

**Keywords:** neurological manifestations, familial hemophagocytic lymphohistiocytosis Type-2, pediatric patients, perforin 1, mutation

## Abstract

Familial hemophagocytic lymphohistiocytosis Type 2 (FHL2) associated central nervous system (CNS) involvement is less understood in children, especially when considering neurologic manifestations as part of the initial presentation. We conducted a retrospective review of the clinical manifestations and genetic abnormality of four Han Chinese children with FHL2 who were patients at the neurology department of Beijing Children’s Hospital from November 2015 to October 2018. These four patients initially manifested CNS symptoms in their disease presentation, and all four patients were misdiagnosed as having ademyelinating disease, such as acute disseminated encephalomyelitis and multiple sclerosis. Given these misdiagnoses, it is important that general physicians and pediatricians maintain awareness of the possibility of FHL2 as a differential diagnosis. These four cases included neurologic manifestations including seizures, ataxia, spasticity, gait disorder, and coma. Bilateral abnormal signals in the cerebrum, including in white matter, gray matter, and junctions were discovered. Enhanced magnetic resonance imaging (MRI) in these patients showed spot or ring enhancement and/or hemorrhage. These patients all possessed a compound heterozygote mutation *PRF1* gene. Whole exome sequencing analysis revealed seven different mutations (three novel mutations) spread over the *PRF1* gene and a heterozygous missense mutation c.1349C > T [p.T450M] that was present in two patients. Three novel mutations, c.634T > C[p.Y212H], c.1083_1094del[p.361_364del], and c.1306G > T [p.D436Y], were discovered and through *in silico* analysis were discovered to be deleterious. Neurologic manifestations were the initial symptoms of FHL2 in these patients in addition to the expected leukopenia and hepatosplenomegaly. Whole exome sequencing of *PRF1* for patients with similar presentations would facilitate prompt and accurate diagnosis and treatment.

## Background

FHL is a severe clinical condition that typically presents with fever, pancytopenia, hepatomegaly, and/or splenomegaly, which can progress to hypertriglyceridemia, hypofibrinogenemia, hepatitis, and/or neurological manifestations (Osinska et al., 2014). Known pathological gene mutations and their associated familial HLH (FHL) subtypes include *PRF1*(FHL2), *UNC13D* (FHL3), *STX11* (FHL4), and *STXBP2*(FHL5) (Dufourcq-Lagelouse et al., 1999; Feldmann et al., 2003). Perforin is the protein product of *PRF1,* and it affects cellular cytotoxicity mechanisms.

Decreased production or activity level of perforin can result in impaired immune defense systems and dysregulation of the apoptotic mechanisms. FHL2 is believed to be invariably fatal during infancy or early childhood unless hematopoietic stem cell transplantation (HSCT) is performed. CNS involvement can be apparent at initial presentation, or it can occur at any time during the course of FHL2. A Chinese single-center study also indicated that twelve patients (13%) had neurological symptoms, including seizures, ataxia, coma, cranial nerve palsy, and hemiplegia (Yang et al., 2010). While the characteristics of FHL2 are well known, FHL2-associated CNS initial involvement is less understood.

We are reporting on four FHL2 cases in which neurological changes appeared as early clinical symptoms. These subjects were inpatients in our neurology department and initially displayed CNS symptoms but had no effects of other systems. They were misdiagnosed as having CNS demyelination prior to having clinical, radiological, and cerebrospinal fluid (CSF) cytology data analyzed. The purpose of this report is to summarize the clinical and genetic characteristics of these cases.

## Case Presentation

We conducted a retrospective review of the clinical manifestations and genetics of four Han Chinese patients with FHL2 at the neurology department of Beijing Children’s Hospital from November 2015 to October 2018.The clinical findings and mutations of the cases are illustrated and listed in **Table 1**.

**Table1:** Clinical Findings and Mutations of *PRF1* in cases.

Patients	Case1	Case2	Case3	Case4
Gender	Female	Female	Female	Male
Age onset	1y 6m	4y 11m	9y	1y 9m
Age diagnosis	12m	14m	37m	2m
Family history	No	Yes	No	No
**Clinical presentation**				
Fever	Yes	Yes	Yes	No
Convulsion	Yes	Yes	Yes	Yes
Splenomegaly	Yes	Yes	No	No
Hepatomegaly	Yes	Yes	No	Yes
Neurological symptoms	gait disorder; seizure and facial paralysis; encephalopathy	headache; seizure; disturbance of consciousness; facial paralysis; ataxia; slurred speech	headache and vomiting and convulsions; ataxia and slurred speech; spasticity; coma	Gait disturbances; convulsion; irritability; somnolence
**Laboratory investigations**				
CSF cytology	10 × 10^6^/L	6 × 10^6^/L	8 × 10^6^/L; 50 × 10^6^/L	4 × 10^6^/L
CSF Protein	850 mg/L	normal	530 mg/L;11200mg/L	684 mg/L
Leukocyte	2.68(10^9^/L)	3.41(10^9^/L)	1.55(10^9^/L)	3.42(10^9^/L)
Neutrophils	0.46(10^9^/L)	0.67(10^9^/L)	0.97(10^9^/L)	0.27(10^9^/L)
Erythrocyte	normal	3.21(10^12^/L)	normal	normal
Thrombocytes	normal	68 (10^9^/L)	normal	normal
Fibrinogen	normal	normal	normal	normal
Triglyceride	normal	normal	normal	normal
Hemophagocytosis in bone marrow smear	No	No	No	No
Ferritin	–	229.4 ng/ml	485.8 ng/ml	–
EBV	negative	negative	CSF EB-IgM positive	negative
*PRF1* paternal origin	c.634T > C	c.1349C > T	c.1349C > T	c.65delC
Protein change	p.Y212Hª	p.T450M	p.T450M	p.P22Rfs*2
*PRF1* maternal origin	c.1083_1094del	c. 853_855del	c.1306G > T	c.148G > A
Protein change	p.361-364delª	p.285delK	p.D436Yª	p.V50M

1.”－”indicated no data.

2. “ª” indicated the novel mutation.

### Case 1

**Figure 1:**
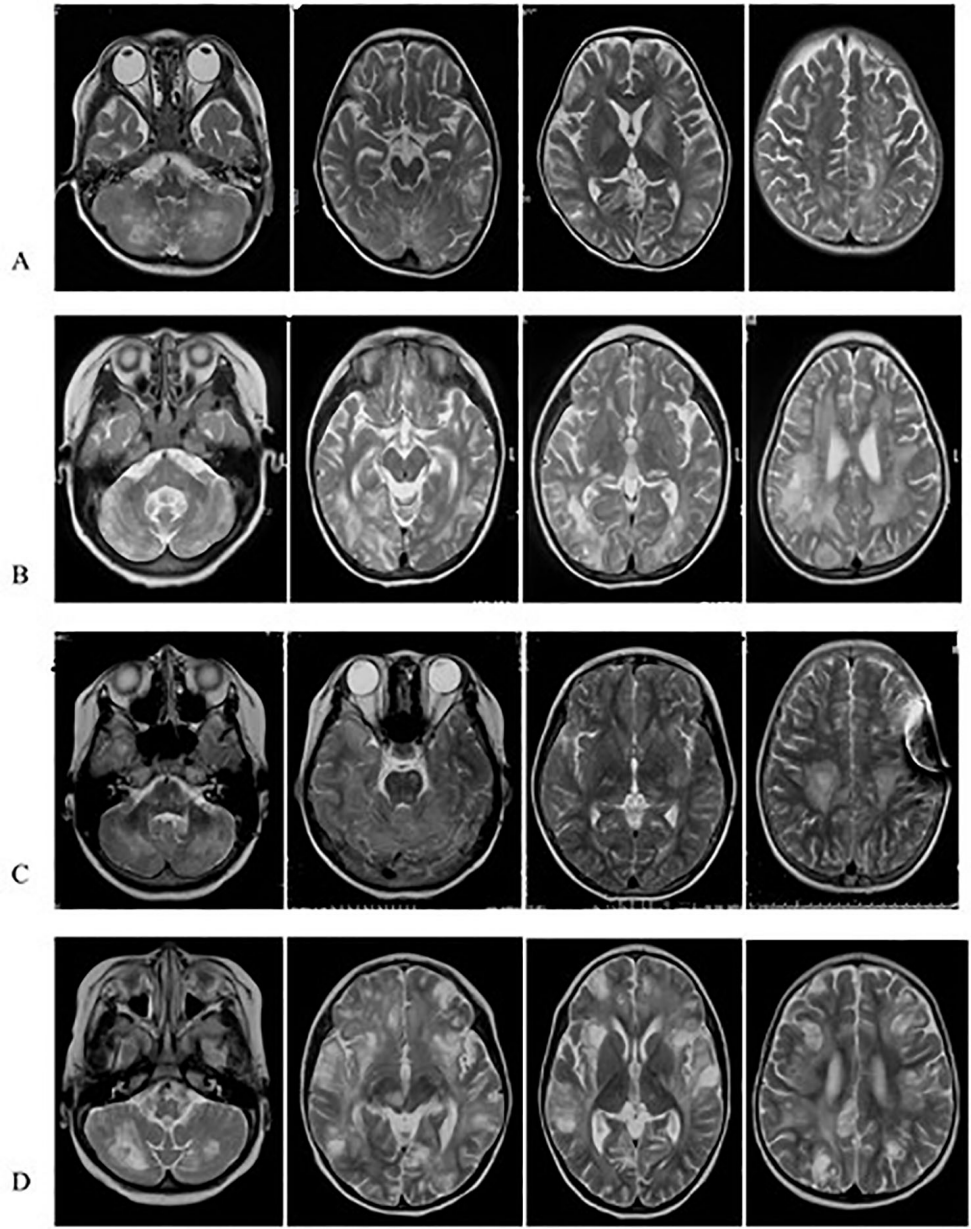
TR-MRIs of Case1 **(A)**, Case2 **(B)**, Case3 **(C)** and Case4 **(D)**. The brain T2-MRIs revealed numerous irregular shape hypersignal areas involving cerebral hemispheres, cerebellar hemisphere, basal ganglia, mostly located in the conticomedullary junction and deep white matter.

**Figure 2:**
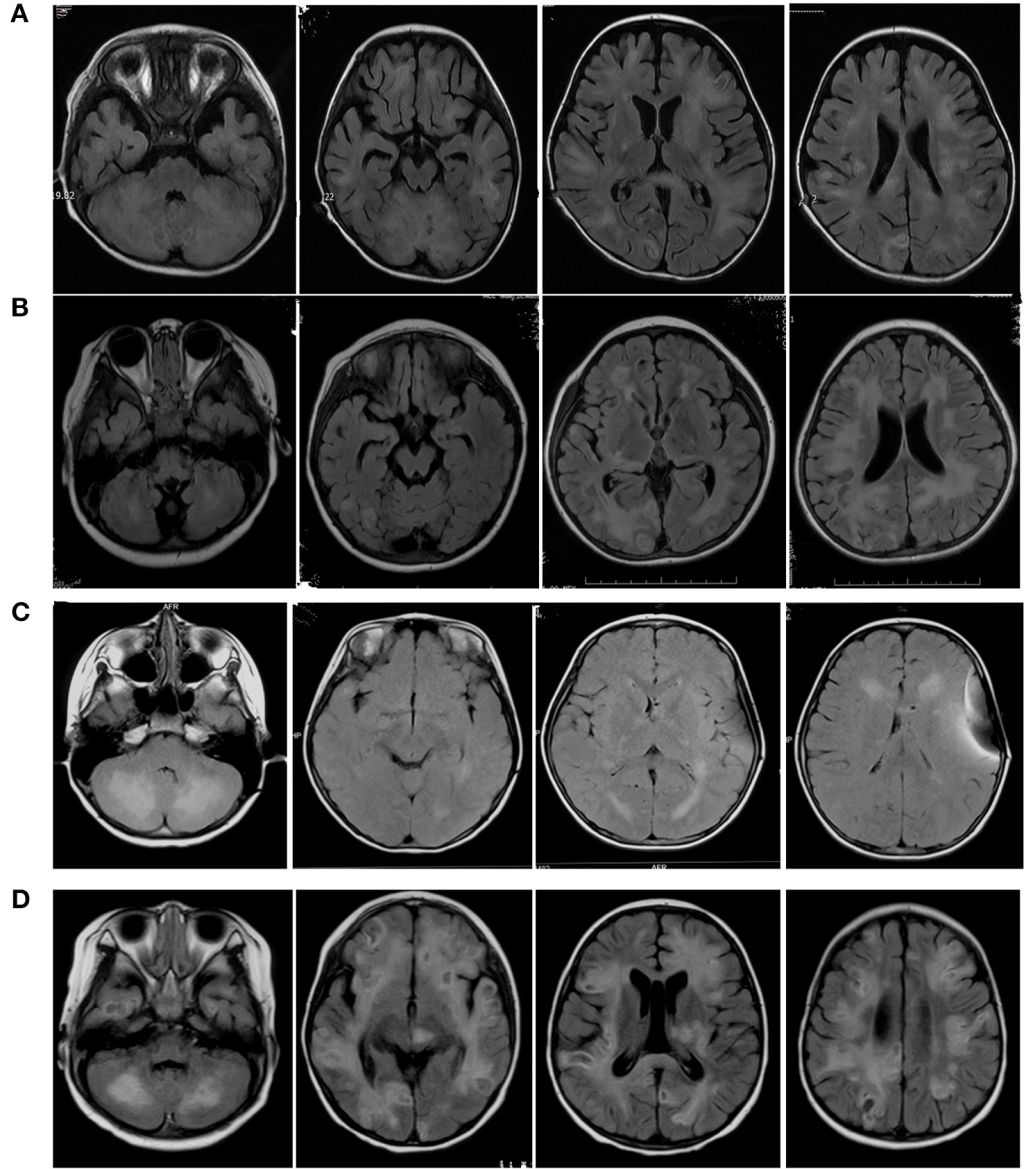
Axial Flair sequences of brain MRIs of Case1 **(A)**, Case2 **(B)**, Case3 **(C)**, Case4 **(D)**. The FLAIR-MRI revealed numerous variable size in the cerebral hemisphere, basal ganglia, cerebellum and brain stem as well as hypersignal intensities on T2-MRI.

In Case 1, an 18-month-old female was admitted to our institution for intermittent fever, weakness, and ataxia over a 15 day period. Brain MRI was performed and multiple irregular lesions were observed in both hemispheres, basal ganglia, and the cerebellum. The focal lesions revealed spot or ring enhancements. The patient developed signs of CNS involvement including seizures and left peripheral facial paralysis. A repeat MRI indicated enlargement of multiple lesions along with an increased number of lesions, and a more defined boundary of abnormal signals was noted in the cerebellum, even though intravenous immunoglobulin (IVIG) and corticosteroids were administered (**Figures 1** and **2**). Abnormalities noted on brain imaging appeared to be roughly proportional to the severity of the clinical manifestations. The laboratory results revealed leukopenia and leukocyte levels at 2.68(10^9^/L) with an absolute neutrophil count of 0.46(10^9^/L). Her condition worsened to include encephalopathy and convulsions. On day 31, she was released from our hospital and admitted to a local hospital. The MRI showed serious encephalatrophy. The girl died from multisystem organ failure 5 months later. About 6 months after Case 1’s death, we diagnosed Case 2, and we observed clinical similarities. We obtained consent from Case 1’s parents and proceeded with whole exome sequencing. The presence of both the c.634T > C[p.Y212H] mutation (a novel mutation of paternal origin) and the c.1083_1094del[p.361_364del] mutation (a novel mutation of maternal origin) confirmed a compound heterozygous state in the subject.

The 2 novel mutations (p.Y212H and p.361-364del) were found to be deleterious *via in silico* analysis by SIFT, PolyPhen and Mutation_Taster software programs. SIFT (http://blocks.fhcrc.org/sift/SIFT.html), which uses evolutionary information from homologous proteins, showed damage, providing a SIFT score 0 (Deleterious sift <= 0.05 ). The PolyPhen tool (http://www.bork.embl-heidelberg.de/PolyPhen/), which incorporates structural information into classification rules, showed probable damage with score of 1 (Probably damaging >= 0.909). The Mutation_Taster (http://www.mutationtaster.org/) prediction indicated “disease causing”. The position of the p.Y212H residue was highly conserved among different species (**Appendix 1A**). The red arrow indicates the difference in the p361-364del PRF1 protein in the three-dimensional model as predicted by the SWISS MODEL (https://swissmodel.expasy.org/) (**Appendix 1B**).

### Case 2

Case 2 was a 4-year-11-month-old female who was admitted to the hospital for symptoms including fever, headache, seizure, and disturbance of consciousness for two days. Primary MRI findings included multiple bilateral abnormal signals in the cerebellar hemispheres and cerebellum, in the posterior extremity of the right inner capsule, in the right brachium pontis, and in the brain stem. Diffusion-weighted imaging (DWI) revealed restricted diffusion in the lesions. Three months later, additional MRI imaging showed scattered, patchy, nodular, and enhanced bilateral abnormal lesions in the cerebrum, cortex, subcortex, periventricular area, basal ganglia, thalamus, midbrain, cerebellum, and pons, in addition to a possible slight hemorrhage without clinical symptoms. Susceptibility-weighted imaging (SWI) showed multifocal low signals. About 6 months after the first hospitalization, she experienced facial paralysis, ataxia, irritability, and slurred speech. Another MRI of the brain indicated enlargement of the lesions (**Figures 1** and **2**). The patient was diagnosed with multiple sclerosis (MS) and prescribed methylprednisolone and IVIG for treatment. The patient then suffered from facial paralysis and gait disturbance. Subsequent brain MRI indicated lesion enlargement and progression. Fourteen months after her first hospitalization, the patient’s younger brother developed leukopenia. The family history included a younger brother with cytopenia of two lineages (platelet and erythrocyte) and hepatosplenomegaly. This information led us to perform whole exome sequencing, which revealed that the patient and her brother carried the same mutations, in *PRF1*, c.1349C > T [p.T450M] (of paternal origin) and c.853_855del [p.285del] (of maternal origin). These two mutations were responsible for FHL2, and the patient and her brother are receiving chemotherapy, awaiting HSCT.

### Case 3

Case 3 was a 12-year-old female admitted to our hospital due to intermittent headache, vomiting, convulsions, ataxia, and slurred speech for three years. Three years prior she was admitted to a local hospital with vomiting and headache. A brain MRI confirmed abnormal lesions in the bilateral cerebrum and cerebellar cortex, abnormal signals in white matter lesions of the cerebellar hemispheres, ventriculomegaly, and cerebellar tonsil herniation. CSF total protein was increased (530 mg/L) and CSF pressure was 300 mmH_2_O. Cell count analysis was normal. The patient was treated for cerebral hernia with external ventricle drainage. The follow-up treatment was ventricle-peritoneal (V-P) shunt operation. During her admission 3 years later, a brain MRI demonstrated new bilateral lesions in the cerebral hemisphere, basal ganglia region, dorsal thalamus, and brainstem (**Figures 1** and **2**). IVIG and corticosteroids were administered to treat MS; however, the patient experienced insufficient symptom relief after treatment. On admission to our hospital, the patient suffered from headache, high fever, and dysphoria. The CSF leukocyte reading was 50 × 10^6^/L and total CSF protein was extremely increased (11,200 mg/L).CSF glucose was low (1.64 mmol/L) and CSF EB-IgM was positive. On the 4th day, her condition deteriorated to a coma with convulsions and respiratory failure. The patient then regained consciousness after several days. Given her three year history and gradual progression, we considered HLH for the differential diagnosis and proceeded with whole exome sequencing. The genetic testing indicated that the patient had two point mutations in *PRF1*, c.1349C > T [p.T450M] (of paternal origin) and c.1306G > T [p.D436Y] (a novel mutation of maternal origin), which was found to be deleterious using *in silico* analysis (these were the same scores seen in Case 1). The p.D436Y position residue was highly conserved among different species.

Five months later the patient could speak but was still unable to walk. A subsequent brain MRI did not indicate any improvement. The patient was receiving chemotherapy in the hematology department of the local hospital.

### Case 4

Case 4 was 21-month-old male who was admitted to our hospital with gait disturbances for 2 weeks. Brain MRI indicated abnormally long T1 and T2 signals in the bilateral left basal ganglia, thalamus, brainstem, and corpus callosum (**Figures 1** and **2**). DWI revealed restricted diffusion within the brain lesions. Enhanced MRI showed scattered, enhanced, and abnormal lesions in the bilateral cerebrum and cerebellum, left basal ganglia, thalamus, brainstem, and corpus callosum. The patient was diagnosed with CNS demyelination, and treated with corticosteroid and IVIG. During the patient’s admission, he suffered from convulsions, irritability, and somnolence. A computed tomography (CT) brain scan displayed lamellar low density bilaterally in the white matter of the cerebrum and cerebellum. Hemorrhagic high density nodules were observed in the temporal and frontal lobes, and mannitol and oxcarbazepine were prescribed as treatment. The patient was discharged in better condition, but with continued gait disturbances. FHL2 was in the differential diagnosis and we proceeded with whole exome sequencing. This confirmed two point mutations in the *PRF1* gene, c.148G > A [p.V50M](of maternal origin) and c.65delC [p.P22Rfs*2] (of paternal origin). The patient is undergoing chemotherapy in our hematology department and is awaiting HSCT.

## Discussion

FHL2 is a rare autosomal-recessive disorder with a poor prognosis, characterized by fever, hepatosplenomegaly, and pancytopenia, which generally presents in infancy or early childhood. In familial cases with a known genetic abnormality (FHL with mutations), a diagnosis can be made without consideration of acquired HLH criteria (Henter et al., 1991; Janka and Schneider, 2004; Henter et al., 2007). In these four cases, the neurologic manifestations were the initial clinical presentation. Three of four cases had fever which was neglected. The expected symptoms such as leukopenia and hepatosplenomegaly appeared as the disease progressed. Laboratory tests revealed leukopenia and neutropenia/agranulocytosis in all four cases. Hepatosplenomegaly was observed in Cases 1 and 2. No patient suffered from hypertriglyceridemia, hypofibrinogenemia, or hemophagocytosis.

The systemic manifestations of FHL typically evolve over a period of time, and neurological manifestations are reported in 20 to 73% of FHL patients (Horne et al., 2008; Dias et al., 2013). These four patients initially manifested CNS symptoms in disease presentation. These neurologic manifestations included seizures, ataxia, facial palsies, spasticity, irritability, gait disorders, and coma. These were consistent with other reports (Henter et al., 1991). Determining a diagnosis is more difficult in patients without a positive family history. In patients with FHL2, neurologic manifestations presenting as the initial clinical indications may delay accurate diagnosis since the symptoms were similar to other neurological diseases, such as acute disseminated encephalomyelitis (ADEM), meningitis, encephalopathy, multiple sclerosis (MS), and CNS vasculitis. All four patients were initially misdiagnosed with a demyelinating disease, such as ADEM or MS. The corticosteroid administration can provide temporary improvement. The time from symptom onset to accurate diagnosis was more than a year in three cases. To identity the clinical features in these cases, the symptoms were often worse than at onset, although transitional symptomatic relief was observed after treatment. Since gray matter dysfunction is relatively common in these patients, all of the patients experienced changes in mental status, described variably as irritability, somnolence, disturbance of consciousness, and encephalopathy. Patients with FHL2 and CNS symptoms often have abnormal CSF findings including mildly elevated cell and/or protein levels. In three cases, the CSF protein content was moderately elevated, although the CSF cell count analysis was normal. The CSF cell count was high in Case 3, which may be associated with a CNS infection.

MRI findings significantly help in the assessment and monitoring of neurological involvement in patients with FHL2 as they reveal abnormal signals in the cerebral hemispheres, basal ganglia, cerebellum, and brain stem as well as hypersignal intensities on T2 and FLAIR images. MRIs also indicate multifocal and bilateral abnormalities with symmetric involvement in T2-weighted imaging. Abnormal signals in the bilateral cerebrum including white and gray matter and junctions were discovered. Abnormal cerebellar lesions were observed in all four cases (**Figures 1** and **2**). Abnormal spot or ring enhancements and/or hemorrhage, especially in the cerebellar hemisphere, were also observed in the brain MRIs (**Appendix 2**). Furthermore, large, ill-defined, confluent lesions were, as the disease was progressive and new lesions were observed subsequent to the original lesion(s). Chronic changes such as atrophy were noted in Case 1. Abnormalities on MRIs appeared to be roughly proportional the severity of the clinical manifestations.

**Figure 3:**
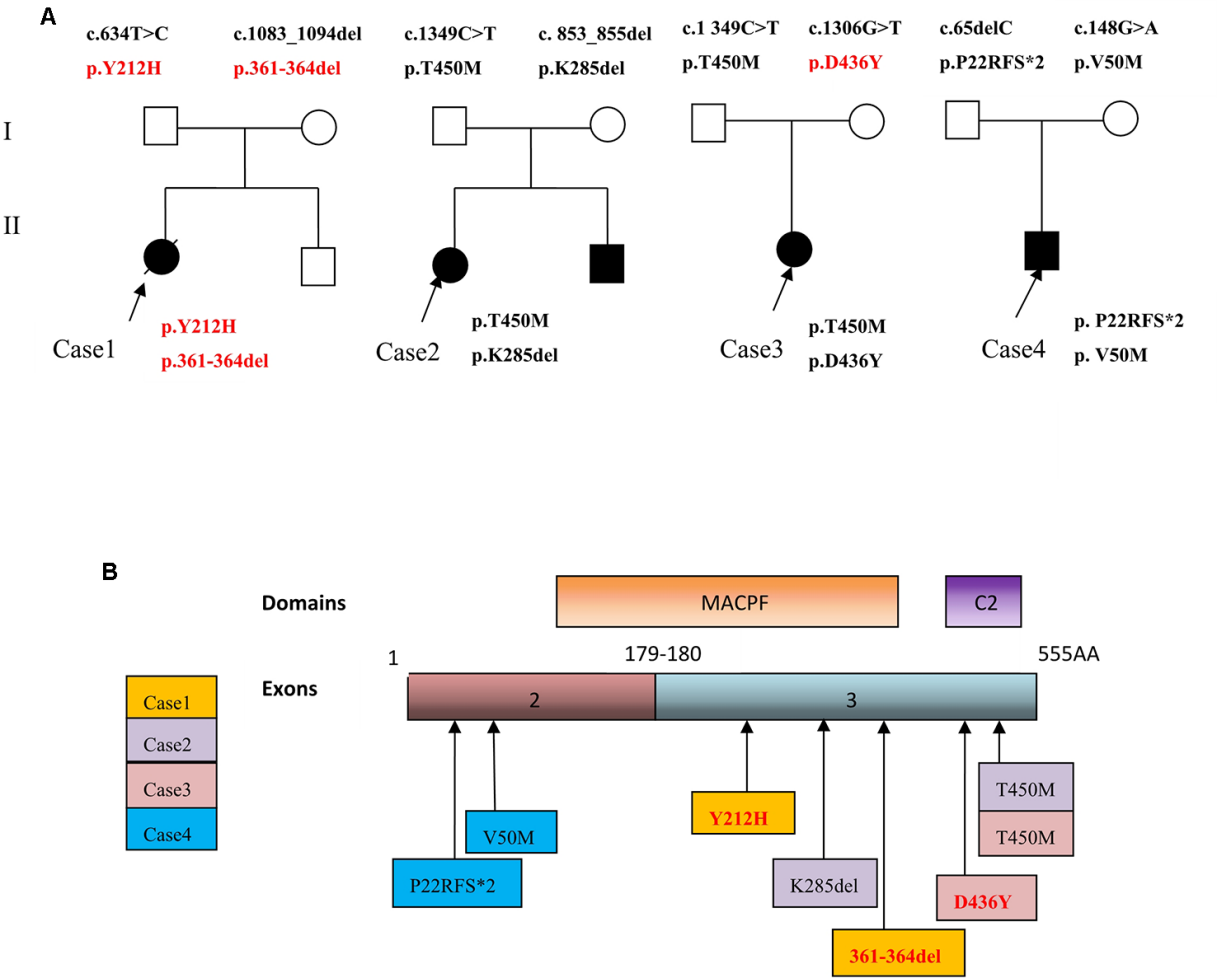
**(A)** Family trees of four FHL2 patients with perforin gene mutations. **(B)** Schematic illustration showing mutation sites of the perforin gene in the FHL2 patients.

In contrast to typical early-onset FHL, our patient initially demonstrated isolated CNS involvement. Nonspecific clinical and neuroradiological findings with initial isolated CNS involvement can result in misdiagnosis and delayed diagnosis in these cases. The diagnosis was established with clinical and genetic testing. An early molecular diagnosis can improve the prognosis of FHL2 patients with prominent CNS involvement. Next-generation gene sequencing greatly aids in diagnosing FHL2. In 1999, *PRF1* gene was first identified as a cause of FHL (Stepp et al., 2015). In four patients, seven different compound heterozygous mutations (of which three were novel mutations) were identified: four missense mutations and three deletion mutations (**Figure 3**). The pathogenic probability of missense mutations was analyzed using different prediction programs. All of these novel mutations were present in the highly conserved region across the species. Cases 2 and 4 had previously reported mutations p.T450M and p.285del; and p.P22Rfs*2 and p.V50M. Cases 1 and 3 had novel mutations p.Y212H, p.361-364del, and p.D436Y. A heterozygous missense mutation c.1349C > T [p.T450M] was present in two patients, Cases 2 and 3.

Case 1 had two novel mutations, which had not been previously published or reported. Ueda *et* al. identified homozygosity for c.1090_1091delCT in exon 3 of the *PRF1* gene, resulting in a frameshift and premature termination (Ueda et al., 2003). This mutation has also been reported in Korea (Kim et al., 2014). In our study, the c.1083_1094del mutation resulted in the loss of the Arg361 Arg362 Ala363 Leu364 [p.361_364del] without a frameshift. These mutations were found to be deleterious *via in silico* analysis. The patient’s condition deteriorated rapidly, and death occurred within several months. This may be due to the mutations, and a further study (such as functional research) may be important.

The mutation in Case 2 c.1349C > T [p.T450M] has also been described in a Japanese case (Ueda et al., 2007) in which a female patient developed HLH at age 7 with an Epstein–Barr Virus (EBV) infection. She had no neurological symptoms; however, brain MRI scans showed high T2 lesions (Ueda et al., 2007). Case2 did not have EBV infection, and the neurologic manifestations were the initial clinical presentation with multiple abnormal signals in the cerebellar hemispheres seen on the MRI. The 3-bp (853_855) deletion in exon 3, resulting in loss of the lys285 residue [c. 853_855del; p.285del] was noted in two reports in two children from Turkey (Goransdotter Ericson et al., 2001; Zur Stadt et al., 2006). For these two patients, no more clinical information was provided, and one patient died before treatment (Goransdotter Ericson et al., 2001).

For Case 3, the patient had two heterozygous mutations, of which c.1349C > T [p.T450M] was also observed in Case 3 as a single heterozygous mutation of paternal origin. This was also reported in two half-brothers in China (Liu et al., 2018). The main clinical manifestations of the older brother were immunodeﬁciency and epilepsy, and CSF analysis showed increased WBCs. The younger brother did not suffer from seizures, but brain effusion cytology showed fewer lymphocytes. An 8-year-5-month-old Chinese girl with recurrent HLH and severe CNS disease was also analyzed with this mutation (Ding et al., 2019). The T450M mutation was also reported in a Chinese study (Zhang et al., 2016) in which the patient was a 2-year-old girl; however, no more clinical information was provided. In our study we discovered two patients with this mutation, which may be an important mutation in China, especially in CNS involvement of FHL2. The c.1306G > T [p.D436Y] is a novel mutation which was determined *via in silico* analysis to be deleterious.

For Case 4, the patient had compound heterozygous mutations. The c.148G > A has been described in a compound heterozygote case in Turkey (Goransdotter Ericson et al., 2001), in which the patient was a 4-month-old female with a fever, splenomegaly cytopenia, and hemophagocytosis, and without CNS symptoms. She had a compound heterozygous mutation in *PRF1*, Tyr219stop, and Val50met and died after the treatment protocol HLH-94 (Henter et al., 1997). The c.148G > A [V50M] missense mutation is conserved in human, mouse and rat (Lowin et al., 1995). The c.65delC mutation [p.P22Rfs*2] was previously reported in two patients from Korea and one from Hong Kong in a compound heterozygous manner (Chiang et al., 2014; Kim et al., 2014; Kim et al., 2017). Two of the patients (one from Korea and one from Hong Kong) were diagnosed with CNS involved FHL2 (Chiang et al., 2014; Kim et al., 2017). The Hong Kong patient’s brain MRI revealed diffuse parenchymal and leptomeningeal enhancing lesions. CSF analysis also showed pleocytosis and a lymphohistiocytic infiltrate. The other Korean patient had progressive multiple organ failure (Kim et al., 2014). The c.65delC[p.Pro22Argfs*2] is a deleterious mutation from the introduction of a premature stop codon due to a frameshift. The three patients reported died after chemotherapy. For our case the boy is undergoing chemotherapy using the HLH−2004 protocol. The long term follow-up is necessary.

The most detrimental *PRF1* mutations associated with minimal or no protein expression, present during early infancy, with a mean onset at 2 months (Trizzino et al., 2008). In our study, four patients with FHL2 were older than one year old at age of onset. One possible reason for this delay is that compound heterozygous *PRF1* missense mutations encode for partially active perforin which might enable patients to survive for a significant period.

Corticosteroid treatment provided improvement in three of four patients; however, Case 1 did not improve, and her diagnosis was made after death *via* whole exome sequencing. There is potentially a strong correlation between genetic defects and the function of perforin. The compound heterozygosity of Case 1 may seriously influence the function of perforin, resulting in death. The severity of the disease depends on the residual activity of perforin. Additional studies could further explain this.

## Conclusions

This study considers neurological manifestations such as seizures, ataxia, and facial palsies, which may provide different clinical presentations than the typical FHL2 symptoms. Thus it is important that pediatricians are aware of the potential diagnosis of FHL2. These symptoms should indicate testing for FHL2 markers, such as fever, leukopenia, hepatosplenomegaly, CSF protein elevation, or brain MRI showing multilobal and widespread lesions in bilateral cerebral hemispheres and the cerebellum. Spot or ring enhancements or hemorrhage were also observed. Whole exome sequencing or genetic analysis of *PRF1* should be performed earlier during the differential diagnosis evaluation to help with prompt, accurate diagnosis, and treatment. Pathogenic mutations in the *PRF1* gene were identified in our patients with FHL2. Three novel mutations were discovered in our study, which may play an important role in the diagnosis of new cases of FHL2.

## Data Availability Statement

The data included in this study are available upon request to the corresponding author of this article.

## Ethics Statement

For research involving human participants, informed consent has been obtained from the patients or the guardian of the patients. The research has been approved by the Ethics Committee of the Beijing Children’s Hospital.

## Author Contributions

Conceived and designed the manuscript: FF, W-XF, C-HD, and T-LH. Clinical data acquisition: W-HZ, J-WL, SG, and X-YY. Analyzed the clinical and genetic data: W-XF and YW. Wrote the paper: W-XF, X-WZ, FF, and YW.

## Funding

This study was funded by the Beijing Municipal administration of Hospitals incubating Program (PX2017065).

## Conflict of Interest

The authors declare that the research was conducted in the absence of any commercial or financial relationships that could be construed as a potential conflict of interest.
