# Supplementary material for: Neurologic Manifestations as Initial Clinical Presentation of Familial Hemophagocytic Lymphohistiocytosis Type2 Due to PRF1 Mutation in Chinese Pediatric Patients
Source: Front Genet. 2020 Mar 4;11:126. doi: 10.3389/fgene.2020.00126 (PMC7064636; doi:10.3389/fgene.2020.00126)
Supplement: Supplementary file 1 [file DataSheet_1.docx]

Homo sapiens **FNASTQPAYLRLISNYG... QDPRREALRRALSQYLTDRA... WGDWFTATDAYVKLFFG**

**D436Y**

**361-364del**

**Y212H**

**species**

Mus musculus **FNSSTEHAYHRLISSYG... QNPKREALRQAISHYIMSRA... WGDYTTATDAYLKVFFG**

Castor canadensis

**FNASTEPDYKMLISTYG... QDPRREALRQALGKYLMGRA... WGDTVTASDAYVKVFFG**

Zalophus californianus

**FNTSTEADYIRLISHYG... WDPRRESLRRAVSKYVMDRA... WGDHFTATDAYLKVFFG**

Cricetulus griseus

**FNTSTKDAYDRLISSYG... QNPRREALRQAISHYVVNKA... WGDIFTASDAYVKVFFG**

Lagenorhynchus obliquidens

**FNISTEPDYWRLISSYG... QDPQREALRQAVSKYVTDRA... WGDLTTATDAYLKVFFG**

Acinonyx jubatus

**FNTSTEADYLRLISNYG... QDPRREALRQAVSKYVMDRA... WGDSFTRTDAYLKVFFG**

**Appendix 1A. The conservation of the novel mutation PRF1 p.Y212H, p.361-364del and p.D436Y**

**
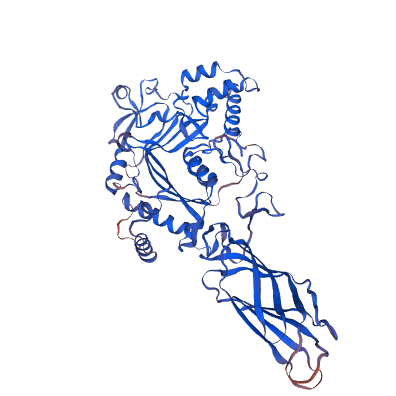
** **
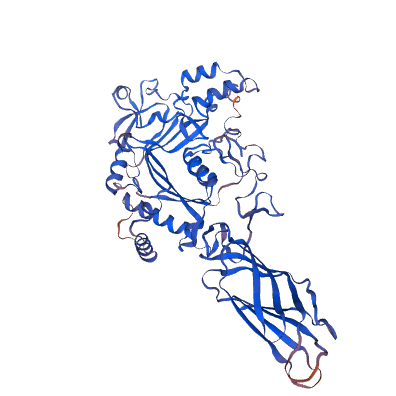
**

**PRF1**

**PRF1p361-364del**

**Appendix 1B. The red arrow indicated the difference of the p361-364del PRF1**

**from the PRF1 protein in the three-dimensional modelling prediction by SWISS MODEL．**
