# Supplementary material for: Neurologic Manifestations as Initial Clinical Presentation of Familial Hemophagocytic Lymphohistiocytosis Type2 Due to PRF1 Mutation in Chinese Pediatric Patients
Source: Front Genet. 2020 Mar 4;11:126. doi: 10.3389/fgene.2020.00126 (PMC7064636; doi:10.3389/fgene.2020.00126)
Supplement: Supplementary file 2 [file DataSheet_2.docx]

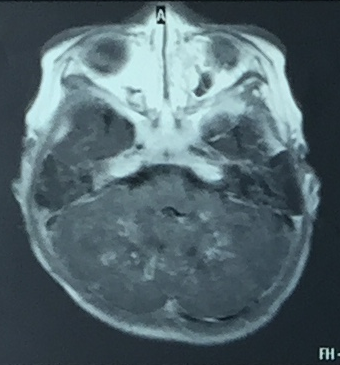

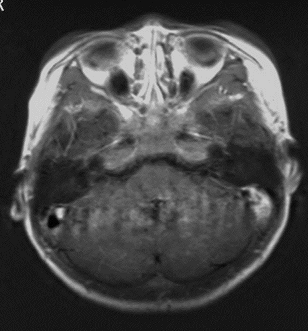


B

A


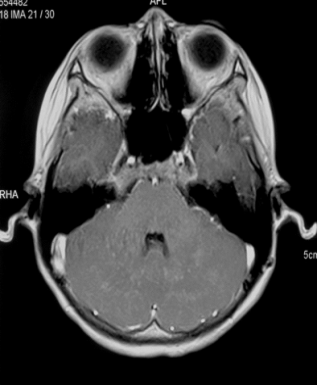

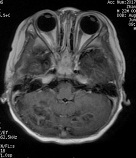


D

C

**Appendix2. The Enhancement MRIs of Case1(A)、Case2(B)、Case3(C)、Case4(D). The Enhancement MRIs revealed spots or ring enhancement in cerebellum.**
